# Interest and Perseverance Are Not Enough to Be Physically Active: The Importance of Self-Efficacy toward Healthy Eating and Healthy Weight to Move More in Adolescents

**DOI:** 10.3390/sports12020041

**Published:** 2024-01-29

**Authors:** María Marentes-Castillo, Isabel Castillo, Inés Tomás, Octavio Álvarez

**Affiliations:** 1 Department of Social Psychology, University of Valencia, 46010 Valencia, Spain; maria.marentes@ext.uv.es (M.M.-C.); octavio.alvarez@uv.es (O.Á.); 2 National Council for Humanities, Science and Technology, Mexico City 03940, Mexico; 3 Department of Methodology of the Behavioral Sciences, University of Valencia, 46010 Valencia, Spain; ines.tomas@uv.es

**Keywords:** grit personality, self-efficacy, healthy eating, healthy weight, physical activity, adolescents

## Abstract

(1) Background: Insufficient physical activity in adolescents remains an important issue for health promotion. Given the current relevance of understanding the adoption and maintenance of moderate and vigorous physical activity (MVPA), the aim of this study was to analyze, in a sample of adolescents, the role of grit personality as an antecedent of healthy eating and healthy weight (HEW) self-efficacy and its implications for the practice of MVPA. (2) Methods: Participants were 987 adolescents (597 girls, 390 boys) aged between 15 and 19 years from Mexico and Spain. The Spanish versions of the grit personality scale, the healthy eating and weight self-efficacy scale and the global physical activity questionnaire were used to measure the variables of interest. (3) Results: Mediated regression analysis showed that grit personality was not directly related to MVPA practice. However, the results indicate the significant relationship between grit personality and HEW self-efficacy, as well as the positive and significant relationship of this self-efficacy on MVPA practice. HEW self-efficacy totally mediated the relationship between grit personality and MVPA in both boys and girls. (4) Conclusions: These results suggest that having a grit personality (i.e., having interest and perseverance) is not enough for adolescents to be physically active, but that perceiving oneself as effective in having a healthy diet and healthy weight may be the key for adolescents to move more. At the intervention level, we suggest targeting an enhancement of young people’s competence to eat healthily and regulate their weight as a strategy to enhance the performance of more MVPA, with a possible transfer between healthy behaviors (spill over).

## 1. Introduction

Physical activity (PA) levels among adolescents aged 11–17 years are insufficient. It is worth noting that adolescent physical inactivity continues to be a significant health promotion issue, not only due to the biological effects of PA [1,2], but also due to its psychosocial effects [3,4]. In school-aged adolescents globally, 80% fail to meet the recommended daily minimum of one hour of PA, with 85% of girls and 78% of boys falling short [5]. Nevertheless, the prevalence of this behavior is not the same in all countries. The current study presents data on adolescents from Mexico and Spain, two countries with high rates of physical inactivity. For example, in Mexico, 42.6% of adolescents aged 15–19 years do not accumulate an average of 60 min per day of moderate to vigorous physical activity (MVPA). A greater percentage of women (49.5%) fail to meet PA guidelines compared with men (34.8%) [6]. In Spain, just 37.7% of 16-year-olds take part in routine PA during their free time. In addition, men (39.7%) aged 15 and over engage in more leisure time PA than women (35.7%) [7]. Related to this issue, the research indicates a substantial reduction in physical activity during the transition from late adolescence to early adulthood [4,8,9]. Spain and Mexico share the same language and many elements of their culture are similar, although there are also important differences between these countries in terms of family and school environment, among others [10]. For this reason, it may be important to examine whether the country has a significant influence on the practice of MVPA.

Numerous studies have analyzed the correlates of physical activity in determining greater PA practice, including gender, age, perceived competence, intention to be active, previous physical activity, social support, and leadership style of physical education teachers, among other factors [11,12,13].

Molina-García et al. [4] found that various psychosocial factors, such as social support and self-efficacy, as well as environmental aspects, such as accessibility to transport of choice, perceived barriers and distance travelled, can influence the level of physical activity undertaken by adolescents. Rhodes and Smith [14] and Reed [15] suggest that personality plays a significant role in the relationship between physical activity (PA) and health practices, due to its strong influence on perceptions, attitudes, norms and self-efficacy, and should therefore be considered when examining PA behaviors [16].

As supported by empirical research conducted by Dunston et al. [17] and Hein et al. [18], one factor associated with physical activity is the personality trait of grit. Grit is a personality trait that prioritizes commitment, interest and sustained effort over time, even in the face of potential obstacles, and includes two dimensions: perseverance of effort, defined as the tendency to work hard and overcome obstacles towards a goal, and consistency of interest, which refers to an individual’s tendency to focus on achieving the same goal over time [19]. Further analysis of the grit personality by Credé et al. [20] suggests that individuals with this trait may initially prioritize perseverance of effort over consistency of interest, leading to greater persistence than consistency in the early stages of development [21], yet this trait remains a predictor of success and performance [22].

In one study, Reed [15] examined the relationship between grit personality and physical activity in a sample of adults and found that grit personality was a significant predictor of the amount and increase of physical activity. Similarly, Hein et al. [18] have found that the intention to be physically active played a moderating role in the effect of perseverance of effort on MVPA in a group of 12–14-year-old children. Hein et al. [23] have demonstrated that perseverance of effort predicts leisure-time physical activity through autonomous motivation in both male and female adolescents aged 11–15 years. Similarly, Dunston et al. [17] and Daniels et al. [24] have revealed a positive association between perseverance of effort and MVPA, as well as home and leisure physical activity in university student samples. Martin et al. [25] established, in a study of adults, that having a grit personality can enhance physical activity levels, decrease time spent sitting, and promote a nutritious diet.

Previous studies have suggested that, to better understand the function of personality traits, it is essential to include other social–cognitive and motivational mechanisms that may mediate the influence of personality on different behaviors, such as the amount of physical activity [26,27]. Self-efficacy, as a social–cognitive mechanism, has been identified as an important correlate of PA [4,12,13] and may also serve as a significant mediator between grit personality and MVPA.

Self-efficacy is conceptualized as a person’s confidence in his or her own abilities to carry out a specific action. Thus, people plan their actions according to the beliefs they hold about their abilities, which can influence the direction, intensity and persistence of their behavior [28,29]. Self-efficacy has been shown to be a variable associated with health-related behavior. In a systematic review, Sheeran et al. [30] found that self-efficacy has a significant impact on health behavior as a mediating and moderating variable. The study has highlighted the way in which self-efficacy can directly influence behavior if the person’s assessment of their efficacy adequately acknowledges their abilities and control over their performance [31,32].

Several studies have explored the relationships between self-efficacy concerning healthy food intake, weight control and PA. Thunfors et al. [33] have found that HEW self-efficacy and physical activity was significantly associated with interest in healthy eating, weight loss, as well as outdoor recreational activities. Olander et al. [34] confirmed the association between improved self-efficacy and increased physical activity through a systematic review and meta-analysis. Gender differences have been observed in relation to health behaviors. Thunfors et al. [33] discovered that men tend to have a greater inclination towards weight training, whereas women are more concerned about weight loss and healthy eating. Additionally, Stephens et al. [35] identified that men, in particular, exhibit lower self-efficacy when it comes to maintaining a healthy diet.

Annesi et al. [36] examined the relationship between physical activity (PA) and healthy eating, highlighting that increases in PA may result from improvements in healthy eating, citing the spillover effect [37,38]. Spillover emphasizes that improvement in one healthy behavior can be transferred to another healthy behavior and/or a psychological aspect such as self-efficacy [39]. Recently, Marentes-Castillo et al. [40] demonstrated in an adult population that initiating weight control can promote increased PA. In fact, Stephens et al. [41], via a systematic review, confirmed that interventions or programs centered on dietary regulation have a stronger impact on weight loss than those that exclusively focus on physical activity.

**Figure 1:**
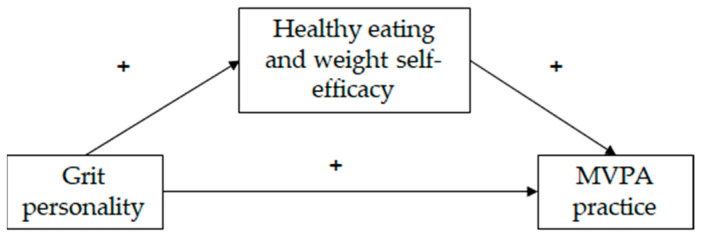
Hypothesized model of the relationships between grit personality, healthy eating and weight self-efficacy and moderate and vigorous physical activity (MVPA) practice. + means positive relationship between the variables.

Given that adolescent physical inactivity remains an important health promotion issue, and the importance of understanding the uptake and maintenance of MVPA practice, this study aimed to test a model that illustrates the relationship between grit personality, self-efficacy in relation to healthy eating and weight management, and the amount of MVPA (see Figure 1) in females and males, controlling for country. The model hypothesizes (H1) that grit personality will be linked to a greater practice of MVPA and (H2) that healthy eating and weight (HEW) self-efficacy will mediate the association between grit personality and MVPA practice. Given the differences between females and males reported in the literature, we will test the hypotheses separately, evaluating the hypothesized model in females and males, controlling for country.

## 2. Materials and Methods

### 2.1. Participants

A total of 987 students from Mexico (*n* = 668) and Spain (*n* = 319) participated in the study, with 597 girls and 390 boys. Their ages ranged between 15 and 19 years (*M* = 16.53; *SD* = 1.18). The participants were selected via a non-probability cluster sampling method from various public schools in the two countries. The inclusion criteria were that the participants must be enrolled with regular attendance in the academic year and be aged between 15 and 19 years.

### 2.2. Instruments

The grit personality scale [19,42] adapted to Mexican Spanish [43] consists of 12 items measuring the following 2 subscales with 6 items each: consistency of interest (e.g., “I often set a goal but later choose to pursue a different one”) and perseverance of effort (e.g., “I have achieved a goal that took years of work”). Responses were collected on a 5-point Likert scale ranging from 1 (not like me at all) to 5 (very much like me). Although the original grit scale was designed to measure two separate grit dimensions, some authors argue that a global grit score can be obtained by combining the two (see Credé et al. [20] for a systematic review), as was undertaken in the current study. The internal consistency value was adequate (Cronbach’s alpha = 0.75).

The healthy eating and weight self-efficacy (HEWSE) scale [44] was elaborated to address an individual’s beliefs that they can engage in behaviors related to healthy food consumption (e.g., “I am able to consume fruits and vegetables in most of my meals”) and weight maintenance (e.g., “If I gain weight, I am able to lose that weight in a timely manner”). Individuals responding to the items of the HEWSE scale are asked to rate their belief about their ability to engage in healthy eating behaviors using a Likert scale that ranges from 1 (strongly disagree) to 5 (strongly agree). This questionnaire was translated and adapted to include Spanish spoken in Mexico and Spain expressions to make it suitable for both samples, following the recommendations of the International Test Commission [45] and using the double translation and alignment procedure. The internal consistency value was adequate (Cronbach’s alpha = 0.89).

Physical activity was assessed with the Spanish version of the global physical activity questionnaire [4]. This questionnaire was developed by the World Health Organization [46] and allows the assessment of MVPA performed during leisure time. This variable was estimated as the product of the frequency (number of days per week) and duration in hours for moderate-and-vigorous-intensity of leisure-time physical activity. Thus, MVPA served to represent the number of hours of practice (moderate and vigorous intensity) per week.

### 2.3. Data Analysis

Descriptive, gender differences (Student’s *t*-test) and correlation analyses between the study variables were performed. In the present study, the percentage of missing data was less than 5% [47], thus it was unlikely to be a serious problem. The association between grit personality and the amount of MVPA practice was tested, as well as the mediating role of HEW self-efficacy in this relationship, using Model 4 in SPSS macro-PROCESS version 3.4.1 [48]. The analysis included the examination of direct and indirect effects between variables and coefficients of determination (R2). The statistical significance was set at 0.05. The significance of the indirect effects (IE) was tested using 95% bootstrap confidence intervals, with 5000 replications [48]. The indirect effects were considered as significant when the confidence interval did not include zero, supporting a mediation effect.

### 2.4. Procedure

This study was conducted in accordance with international ethical guidelines consistent with the American Psychological Association and the Declaration of Helsinki. All procedures involving participants in the research study were approved by the Commission on Ethics in Experimental Research of the University of Valencia (Ref: 1707311). The data collection involved four stages. In the first stage, 9 schools in Mexico and 12 schools in Spain were contacted, a request was sent to them for permission to collect data online from adolescents between 15 and 19 years of age who were active in the school year. All schools agreed to participate in the study. In stage 2, once informed consent was collected, the online survey was distributed to all school participants. In stage 3, after the survey was closed, the 9 schools in Mexico participated with 769 students, and in Spain only 9 schools participated with a total of 328 students, for a total of 1097 students. Finally, in stage 4, in which we applied the inclusion and exclusion criteria, we analyzed 987 participants for the present study. The data collection was carried out during the months of March to June 2022, using online google forms. Prior to data collection, a real-time connection was made in order to explain to the participants the procedure to be followed, together with the presence of the teacher in charge of the school group. Participants were informed of their consent to participate freely and anonymously in the study before spending 15–20 min filling in the online forms.

## 3. Results

Table 1 shows the descriptive values for girls and boys, gender differences and bivariate correlations between the different variables by gender. Regarding the academic level, 732 (74.2%) participants were from high school, 139 (14.1%) from secondary education (ESO), 108 (10.9%) attending their first year of university and 8 (0.8%) from professional formation (FP). Independent-sample t-tests indicated similar mean values for grit personality and HEW self-efficacy across gender (Cohen’s d = 0.04 and 0.12 for grit personality and HEW self-efficacy, respectively, indicating non-relevant differences) but different values for the number of MVPA sessions. This difference confirmed that boys do more MVPA per week than girls (Cohen’s d = 0.30, indicating small differences). All variables were significantly positively correlated with each other for both girls and boys.

**Figure 2:**
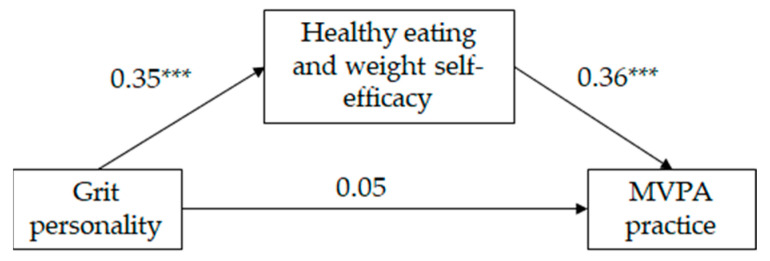
Standardized solution of the hypothesized model of the relationships between grit personality, healthy eating and weight self-efficacy and moderate and vigorous physical activity (MVPA) for girls (*n* = 597). *** *p* = 0.001.

**Figure 3:**
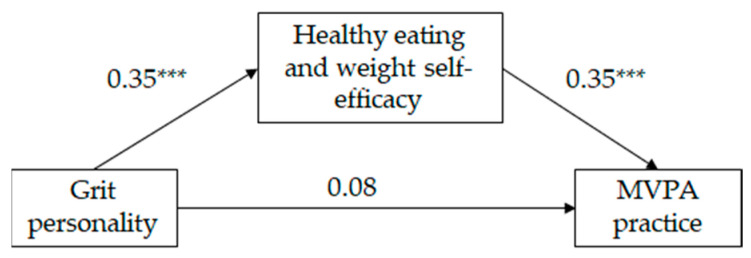
Standardized solution of the hypothesized model of the relationships between grit personality, healthy eating and weight self-efficacy and moderate and vigorous physical activity (MVPA) for boys (*n* = 390). *** *p* = 0.001.

As can be seen on Table 2, and in Figure 2 and Figure 3, the mediated regression analysis showed that, for both girls and boys, grit personality was positively correlated with HEW self-efficacy and HEW self-efficacy was positively correlated with MVPA practice (following Cohen [49], the effect size of the aforementioned relationships was medium). The direct relationship between grit personality and MVPA practice was significant for neither girls neither nor for boys; however, the indirect relationship between grit personality and MVPA practice, through HEW self-efficacy, was significant (IE = 0.12, SE = 0.02, LLCI = 0.09; ULCI = 0.17 for girls and IE = 0.12, SE = 0.02, LLCI = 0.08; ULCI = 0.17 for boys). Following Wen and Fan’s [50] recommendations, we estimated P_M_ coefficient as an effect size measure of the indirect effect. Results indicate that 72% and 60% (for girls and boys, respectively) of the total effect of grit personality on MVPA practice was mediated by HEW self-efficacy. The model for girls explained 12% of the variance in HEW self-efficacy and 16% of the variance in MVPA practice. The model for boys explained 12% of the variance in HEW self-efficacy and 15% of the variance in MVPA practice. Country did not show a significant effect on HEW self-efficacy on either gender, but did show a significant effect on MVPA practice for girls only. Mexican girls practice more MVPA than Spanish girls.

## 4. Discussion

The aim of this study was to test a model of the relationships between grit personality, self-efficacy to engage in healthy eating and maintain a healthy weight, and the amount of MVPA in Mexican and Spanish boys and girls, controlling for country.

In terms of descriptive data and differences, we observed significant differences in the amount of MVPA in relation to gender. Boys performed more MVPA than girls. These results confirm the findings reported by Medina et al. [6] in Mexico and in Spain [7], showing that it is boys who continue to report a higher amount of PA. In this sense, Thunfors et al. [33] mentioned that males are more interested in bodybuilding, which is likely to lead them to engage in more PA, whereas females are more interested in losing weight and eating healthily.

Two hypotheses were put forward in this study, the first of which (H1) states that grit personality will be associated with a greater practice of MVPA. We tested this hypothesis in boys and girls, controlling for country. Our findings support this hypothesis, as we found that the relationship between grit and MVPA practice was significant for both genders. These results are consistent with those described by Rhodes and Smith [14] and Reed [15], who confirmed the positive effect of grit personality on the amount of PA performed. However, it should be noted that these studies were conducted with an adult population, so our study adds empirical evidence for this relationship also with adolescents. Additionally, the benefits of physical activity in children and youth can lead to the strengthening of the grit personality in adulthood [51].

In the second hypothesis (H2), we assumed that HEW self-efficacy would mediate the relationship between grit personality and MVPA practice. Our results support this hypothesis, as we found that HEW self-efficacy fully mediated the relationship between grit personality and MVPA practice for both girls and boys. These results are consistent with those of Hein et al. [18,23], who focused only in one dimension of the grit personality, perseverance of effort, finding that this dimension indirectly influenced PA practice through different mediators, such as intention to be physically active and autonomous motivation. These findings could be related to the analysis of Credé et al. [20], who stated that people with this grit trait may develop perseverance of effort before consistency of interest, which is more common in early stages such as adolescence [21]. This may explain why grit personality, as measured by its two global indicators, has no specific impact on PA practice among young people.

On the other hand, although self-efficacy has been reported as an important correlate of PA [4,12] and as a variable associated with health behavior [30], to our knowledge, HEW self-efficacy has not been used as a mediating variable in the relationship between grit and MVPA practice. These findings are also in line with Thunfors et al. [33] and Olander et al. [34], who found that self-efficacy towards healthy consumption was associated with outdoor recreational activities and performing more PA.

Our results not only confirm that self-efficacy is an associated and mediating variable towards PA practice, but also highlight the interaction between healthy behaviors, such as the spillover effect, which has recently been studied and shows that weight regulation behaviors, and in this case HEW self-efficacy, can lead a person to perform more PA [39,40,41].

The renewed interest in studying the role of personality, and grit personality in particular, in PA [15] emphasizes that, while grit personality may be beneficial in increasing PA levels [16,25], it needs to be studied through other psychosocial mechanisms [26,27]. In this case, our study provides evidence that HEW self-efficacy may be an important variable when promoting greater PA in adolescents. It is important to remember that for HEW self-efficacy to mediate PA practice, adolescents must have the necessary skills and competencies to engage in healthy consumption and healthy weight maintenance. Adequate assessment of these skills and competencies will lead them to have better control over their performance [29,31] in healthier consumption choices and weight regulation. Similarly, studies that have studied grit personality and physical activity have shown that this personality trait can predict youth leisure-time physical activity over time but through another psychosocial mechanism such as autonomous motivation [23].

It is necessary to understand the practice of PA in adolescence, given the abundance of data on the decline of PA in this developmental stage and the physical inactivity that persists in this stage of life [1,6]. This highlights the way in which, in adolescence, it is not only important to move, but it is essential to engage in moderate and vigorous physical activity in the recommended amounts [52].

As recommendations for intervention, it is important to train young people in healthy consumption and weight regulation through schools and with the involvement of health professionals. Training in healthier consumption and the importance of weight regulation will help to increase their confidence in their ability to adopt healthy behaviors in the contexts in which they operate. Having greater control over their own behavior through self-efficacy will lead them to be able to manage social and environmental factors (such as the advertising of unhealthy products) and to recognize the importance of staying physically active in order to regulate weight in a healthy way.

However, we know that the role of the family will be essential for the development of self-efficacy towards healthy consumption and weight regulation, so, as a proposal for study and intervention, we aim to explore competencies in terms of eating that are held in the family nucleus, and which in turn will be a learning model for young people. In addition, it would be interesting to develop future studies considering the two sub-factors of grit personality (i.e., perseverance of effort and consistency of interest) and also studies including adult samples. Future studies should also include other factors that might influence PA levels, such as other personality traits (e.g., perfectionism), culture, customs, education level, publicity effects, environment variables (e.g., social support, motivational climate, perceived barriers), economic resources, technological facilities, eating customs, etc. The limitations of the study include the cross-sectional nature of the research, which does not allow us to establish causal conclusions. In addition, there is a need for more information on the PA carried out by the young people surveyed (e.g., motives for undertaking it, and other socio-demographic variables).

## 5. Conclusions

Conclusions of the study include the importance of including the role of grit in health studies and investigating its influence through psychosocial mechanisms, such as self-efficacy for healthy eating and weight regulation, in order to promote greater levels of MVPA. If young people know more about the control they can have over their diet and weight, they will be interested in increasing the amount and intensity of their physical activity. It is becoming increasingly clear from these studies that the key to ensuring that people do more PA and make healthy choices lies in psychosocial mechanisms, rather than simply recommending more PA, better diet and weight control. In summary, interest and perseverance are not enough for adolescents to be physically active. To encourage them to be more active, it is important to consider how effective young people think they are at managing their diet and weight in a healthy way.

## Figures and Tables

**Table 1:** Descriptive statistics, gender differences and correlations between the study variables.

	Girls (*n* = 597)		Boys (*n* = 390)					
Variables	*Mean*	*SD*	*Mean*	*SD*	t	1	2	3
1. Grit personality	3.20	0.64	3.17	0.57	0.66	-	0.35 **	0.18 **
2. HEW self-efficacy	3.40	0.88	3.50	0.80	−1.91	0.34 **	-	0.38 **
3. MVPA	5.37	6.57	7.50	7.72	−4.49 **	0.20 **	0.38 **	-

Note. Ranges of HEW self-efficacy and grit personality 1–5, range of MVPA 0–39. HEW = healthy eating and weight and MVPA = moderate and vigorous physical activity. Girls’ values are above the diagonal. Boys’ values are below the diagonal. ** *p* < 0.001.

**Table 2:** Results of the mediation models by gender.

	HEW Self-Efficacy		MVPA Practice	
Predictors	B	t	B	t
Girls (*n* = 597)				
Grit personality	0.35	8.98 ***	0.05	1.30
HEW self-efficacy			0.36	8.96 ***
Country	−0.06	−1.49	−0.11	−2.99 ***
F	41.57 ***		38.37 ***	
Boys (*n* = 390)				
Grit personality	0.35	7.23 ***	0.08	1.61
HEW self-efficacy			0.35	7.03 ***
Country	0.03	0.72	−0.05	−1.05
F	26.19 ***		22.76 ***	

*Note*. HEW = Healthy eating and weight, MVPA = moderate and vigorous physical activity. *** *p* = 0.001.

## Data Availability

All data used in this study are presented in the manuscript.
